# Characteristics of the Structure, Mechanical, and Tribological Properties of a Mo-Mo_2_N Nanocomposite Coating Deposited on the Ti6Al4V Alloy by Magnetron Sputtering

**DOI:** 10.3390/ma14226819

**Published:** 2021-11-11

**Authors:** Stanisław Adamiak, Wojciech Bochnowski, Andrzej Dziedzic, Łukasz Szyller, Dominik Adamiak

**Affiliations:** 1 College of Natural Sciences, University of Rzeszow, Pigonia 1, 35-310 Rzeszow, Poland; dziedzic@ur.edu.pl; 2 PlasmaVac, Unit 6 Barnack Ind Estate, Salisbury SP2 0AW, UK; lukszy83@gmail.com; 3 Mechanical and Industrial Engineering, University of Toronto, 5 King’s College Road, Toronto, ON M5S 3G8, Canada; dominik4a@gmail.com

**Keywords:** Mo-Mo_2_N, coatings, nano-structure, mechanical properties, tribological wear, magnetron sputter

## Abstract

Mo-Mo_2_N nanocomposite coating was produced by reactive magnetron sputtering of a molybdenum target, in the atmosphere, of Ar and N_2_ gases. Coating was deposited on Ti6Al4V titanium alloy. Presented are the results of analysis of the XRD crystal structure, microscopic SEM, TEM and AFM analysis, measurements of hardness, Young’s modulus, and adhesion. Coating consisted of α-Mo phase, constituting the matrix, and γ-Mo_2_N reinforcing phase, which had columnar structure. The size of crystallite phases averaged 20.4 nm for the Mo phase and 14.1 nm for the Mo_2_N phase. Increasing nitrogen flow rate leads to the fragmentation of the columnar grains and increased hardness from 22.3 GPa to 27.5 GPa. The resulting coating has a low Young’s modulus of 230 GPa to 240 GPa. Measurements of hardness and Young’s modulus were carried out using the nanoindentation method. Friction coefficient and tribological wear of the coatings were determined with a tribometer, using the multi-cycle oscillation method. Among tested coatings, the lowest friction coefficient was 0.3 and wear coefficient was 10 × 10^−16^ m^3^/N∙m. In addition, this coating has an average surface roughness of RMS < 2.4 nm, determined using AFM tests, as well as a good adhesion to the substrate. The dominant wear mechanism of the Mo-Mo_2_N coatings was abrasive wear and wear by oxidation. The Mo-Mo_2_N coating produced in this work is a prospective material for the elements of machines and devices operating in dry friction conditions.

## 1. Introduction

Titanium and its alloys are widely used in the aerospace, automotive, chemical, energy, and biomedical engineering industries. The decisive factors for the use of titanium alloys are: high specific strength, good fatigue properties, high corrosion resistance, and biocompatibility. The Ti6Al4V alloy (ASTM Grade 5) is the most commonly used two-phase (α + β) alloy, in which the addition of Al (6.5 wt.%) strengthens the α phase, while the β phase is stabilized by introduction of isomorphic V (Vanadium) (3.5 wt.%) into the alloy.

However, the relatively low hardness of titanium, low modulus of longitudinal elasticity, high chemical activity at elevated temperatures, and a tendency to build up and stick to mating surfaces [1,2] mean that titanium and its alloys have only limited uses under tribological wear conditions. For the above reasons, the surface modification of titanium alloys is still an active research subject for industrial applications. Technologies for the surface treatment of titanium alloys, which include nitriding, nitrogen ion implantation [3], and physical vapor deposition (PVD) enable the production of metal nitride coatings that exhibit increased tribological resistance [4,5].

Among the PVD techniques, cathodic and magnetron sputtering are often used to coat metal surfaces. Among the coatings deposited on the metallic substrates, the ones consisting of titanium, chromium, zirconium, molybdenum, and tantalum nitrides (characterized by high hardness, high tribological wear resistance, and good adhesion to the substrate) are widely used [6,7,8,9]. The addition of aluminum preserves the hardness at elevated temperatures [10]. Monolithic single and multi-component molybdenum nitrides are characterized by high hardness, good chemical stability, and a high melting point. Thin Mo_2_N layers retain their properties up to 500 °C [11].

Moreover, the molybdenum contained in these coatings has the ability to form oxides that act as a lubricant to effectively reduce tribological wear. It is assumed that the low friction coefficient of approximately 0.3 [12,13] for coatings containing Mo is associated with the formation of the Magneli MoO_3_ phase during the friction process, which is characterized by favorable sliding properties [14,15].

The crystallization of the coating, its structure, and phase stoichiometry, as well as the orientation of the crystallites, is strongly correlated with the parameters of the deposition process. Many publications describe the influence of the deposition parameters on the physical and mechanical properties of coatings. Regardless of the investigated property, in comparison to other process parameters, the dominant effect of nitrogen concentration in the PVD chamber can be observed. Depending on the production parameters, the dominant phase of the Mo-N coating may be the: α-Mo supersaturated with nitrogen (with a spatially-centered regular structure), γ-Mo_2_N (with a wall-centered regular structure), β-Mo_2_N, non-equilibrium β′-Mo_2_N, β″-Mo_2_N phases (with wall-centered tetragonal lattice), and δ-MoN hexagonal phase [16,17,18]. By controlling the parameters of the deposition process (nitrogen pressure, polarization voltage, and substrate temperature) it is possible to optimize the share of each of the δ–MoN, γ–Mo_2_N phases, in order to achieve high hardness of the coatings [19,20]. The bias voltage can be used to control the growth direction of the molybdenum nitrides, which grow as columnar crystals. It is also possible to obtain an amorphous structure of molybdenum nitride coatings, with a small proportion of nanocrystallites, through an increase in nitrogen concentration in the reaction chamber [11]. According to Bouaouin et al. [21], the phase composition of the thin layer influences the morphology, stress state, and mechanical properties. The molybdenum nitride coating exhibits a polycrystalline structure, with preferred orientation along the plane (111). An increase in the nitrogen content in the coating causes a decrease in the size of crystallites.

Coating hardness and Young’s modulus in Mo-N coatings varies from 22 GPa to 33 GPa [16,20,22,23] and from 260 GPa to 400 GPa [10,22,23,24], respectively, depending on the process method and parameters. High values of the H/E > 0.1 ratio ensure favorable tribological properties [25]. Hard monolithic coatings have low fracture resistance, leading to premature failure. The most successful strategies to improve the fracture resistance of ceramics are based on the production of multilayer [9,26,27] and nanocomposite coatings [28,29]. The H^3^/E^2^ ratio is an important parameter that allows the classification of coatings, in terms of resistance to plastic deformation. In the case of multilayer CrN/MoN coatings [30], a dependence of the internal structure, and thus the mechanical properties, on the thickness of individual layers of the coating, were found. A significant increase in the hardness and impact toughness was observed, along with a decrease in the thickness of individual layers to 20 nm: H = 38–42 GPa, H/E = 0.11.

A novelty of this work is the production of Mo-Mo_2_N nanocomposite coating, deposited on Ti6Al4V alloy and characterized by low tribological wear. To the authors’ knowledge, there is no research work in the available literature on Mo-Mo_2_N composite deposited on Ti6Al4V alloy. Earlier works were related to a single-phase Mo_2_N, MoN, or multi-phase Mo_2_N/MoN coatings deposited on metallic substrates. 

The main goal of this work was to obtain a nanocomposite with low friction coefficient and tribological wear, which was formed by nanocrystallites of the Mo matrix and nanocrystallites of the reinforcing phase of the Mo_2_N nitride. The coating was produced on Ti6Al4V alloy by a reactive magnetron sputtering process at various nitrogen flow rates.

## 2. Materials and Methods

The research materials were the above-mentioned Mo-Mo_2_N nanocomposite coatings. The coatings were deposited on a Ti6Al4V alloy substrate with dimensions of 25 mm × 25 mm × 0.5 mm and roughness Ra = 5 nm. Prior to coating formation, the substrates were cleaned with ethyl alcohol in ultrasonic bath. Coatings were produced using PREVAC (Company Ltd., Rogow, Poland) equipment by physical vapor deposition using a DC reactive magnetron sputtering process.

A molybdenum target of 25.4 mm in diameter with a purity of 99.99 wt.% made by Kurt J. Lesker (Company Ltd, St Leonards On Sea, East Sasex, United Kingdom) was used during sputtering process. Distance of the target from the substrate was 70 mm. Argon 6.0 was used as the working gas and nitrogen 5.0 was the reactive gas. The Ti6Al4V substrates were placed centrally to the target to ensure sufficient uniformity of the deposited film without the need to rotate the substrate. Sputtering was performed under conditions of nominally unheated substrate. However, during the deposition, the substrate and holder heated up to about 80 °C by self-heating as a result of bombardment of the surface with argon ions. In order to improve adhesion of the Mo-Mo_2_N nanocomposite coating to the substrate, in the first step, a transition layer was created by gradually increasing nitrogen flow rate from 0 to a preset value over a period of 360 s. Then, the preset nitrogen flow was maintained for the duration of coating formation. Magnetron power was kept constant at 70 W. Parameters of the Mo-Mo_2_N coating production process are presented in Table 1.

Crystal structure of the coatings was determined using Bruker D8 Advance X-ray diffractometer (Bruker Corporation, Billerica, MA, USA. The source of the X-ray radiation was the Cu-Kα lamp. In order to limit the intensity of the peaks coming from the substrate, the GID (Grazing Incidence Diffraction) method was used for the angle ω = 3°. Measurements were made in the angular range of 2θ = 20–70° with scanning speed of 0.01°/s and resolution of 2θ = 0.015°. Phases present in the structure were identified on the basis of patterns from the ICDD PDF database: 00-001-1208 (Mo), 00-025-1366 (γ-Mo_2_N), using Bruker EVA program (ver. 5.2, PDF 2.1, Bruker Corporation, Billerica, MA, USA. Metallographic tests were carried out using electron microscopy. Using an SEM/FIB electron microscope Quanta 3D 200i (FEI Company, Eindhoven, Netherlands), microstructures of the surface of the coatings and sections perpendicular to the coating were observed. During the analysis, SE and BSE detectors were used. The analysis of the chemical composition EDS was performed using Octane Elect system. Structure of the coatings was analyzed using a Tecnai Osiris (200 kV) transmission electron microscope (FEI Company, Eindhoven, Netherlands) with the use of HAADF and BF detectors and electron diffraction from selected areas (SAED). Fourier transformation was used in the analysis of the high-resolution images.

Microanalysis of chemical composition and mapping of the distribution of elements were performed with an energy dispersion spectrometer (TEM with EDS, FEI Company, Eindhoven, Netherlands) in nano-regions. Measurements of coating roughness were performed using an AFM-CSM atomic force microscope in contact mode.

Measurements of hardness and Young’s modulus were carried out using nanoindentation method on a CSM stand, according to the ISO 14577 1:2015 09 standard [31]. Young’s modulus of the tested coating was calculated based on the method of Oliver and Pharr [32]. Force acting on the indenter was 5 mN, operating time was 15 s, and the rate of force increase was 1.66 mN/s. The maximum value of the loading force was selected so that the maximum impression depth was less than 10% of the coating thickness. Hardness was calculated as the arithmetic mean of 7 measurements.

Adhesion of the coating to the substrate was determined by the scratch test using a Micro Combi Module MCT-CSM. A Rockwell indenter with radius of 100 µm was used, the maximum force was 30 N, the rate of the force increase was 10 N/min and the length of the scratch was 3 mm. The critical loads Lc_1_, Lc_2_, Lc_3_ were determined on the basis of recorded friction force, acoustic emission signal and scratch observation with the SEM electron microscope. Three scratches were made in each coating and the results were averaged.

Tribological properties of the coatings were determined with a tribometer ((TRN, CSM Instruments, Peseux, Switzerland) using the multi-cycle oscillation method. Tests were carried out using linear reciprocating motion, in which the sample moved against a stationary ball with travel speed of 0.02 m/s. Length of path for one friction cycle was 6 mm. Wear of the coating material was determined after 10,000 cycles, which corresponded to a total friction distance of 60 m. As a counter-sample, a ceramic Al_2_O_3_ ball with diameter of 6 mm was used. The ball was loaded with normal force of 1 N, 2 N and 5 N. Measurements were carried out at room temperature and 55% relative air humidity.

## 3. Result and Discussion

### 3.1. Chemical Composition of Coatings

In order to determine influence of nitrogen flow rate on its content in the coating, chemical composition tests were carried out with the use of an EDS microanalyzer. As the nitrogen flow in the reaction chamber increased, nitrogen content in the coating increased. Nitrogen concentration in the coating varied from 5.9 at.% for the nitrogen flow rate of 0.20 sccm to 18.6 at.% for the nitrogen flow rate of 0.60 sccm. Increasing nitrogen flow rate by 0.2 sccm increases its concentration in the coating by an average of 6 at.%. Increasing quantity of nitrogen in the reaction chamber also reduces the deposition rate of the coating. Thickness of the coatings ranged from 0.98 µm to 1.33 µm (Table 2).

### 3.2. Phase Composition of Coatings

**Figure 1:**
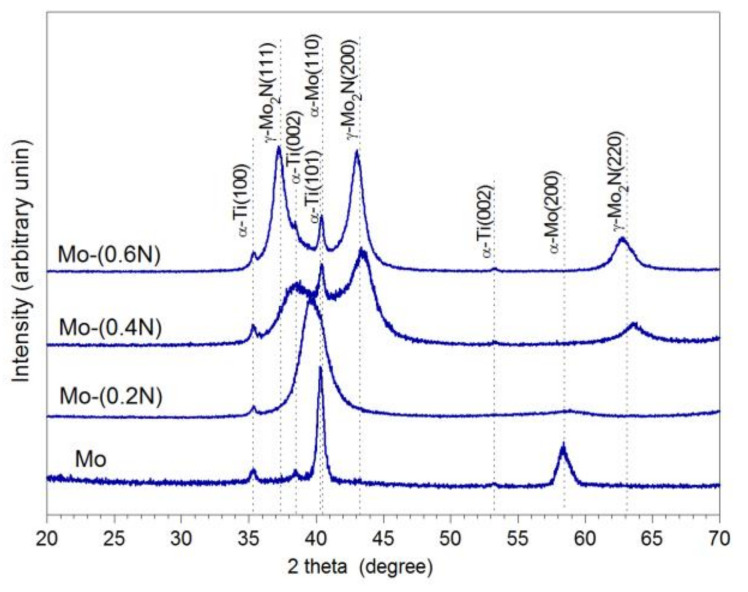
XRD diffractogram of Mo-Mo_2_N nanocomposite coatings.

In order to identify phase composition of the produced coatings, XRD tests were carried out. Peaks measured from the Mo sample agree very well with the data for the body-centered cubic α-M included in PDF-4 for the planes (110) and (200) respectively. Broadening of the peaks, especially (200), is related to the significant fragmentation of the structure formed in the magnetron sputtering process. Despite adopted angle of incidence of the beam ω = 3°, diffraction pattern also showed peaks from the Ti6Al4V substrate, very well aligned with data for α-Ti. After introduction of nitrogen (7% in the Ar + N_2_ gas mixture), the diffraction patterns of the Mo-(0.2N) sample showed extended and displaced peaks, based on Mo. Intensity of the α-Mo (100) peak remained at a comparable level, while the α-Mo (220) peak disappeared (Figure 1). According to the equilibrium system [11], nitrogen does not dissolve in molybdenum at low temperatures.

Only at 1800 °C does nitrogen dissolve in molybdenum to the maximum amount of 1.08 at%. In the Mo-(0.2N) coating, average nitrogen concentration was 5.9 at.%. Under non-equilibrium conditions of the PVD process, as the result of incorporating nitrogen in interstitial states, a supersaturated α-Mo solid solution is formed, highly refined, almost amorphous. The average size of crystallites in the Mo coating was 18.5 nm and in the Mo(0.2N) coating it was 2.4 nm (Table 2). According to Bragg’s equation, the shift of the α-Mo (110) peak towards lower angular values observed in the Mo-(0.2N) coating corresponds to increased parameters of the crystal lattice. Broadening and shift of the α-Mo (110) peak, resulting from nitrogen incorporation, was also demonstrated in [17], with a higher 10% share of N_2_ in the Ar + N_2_ mixture.

Increasing nitrogen flow to 0.4 sccm leads to the formation of a cubic molybdenum nitride phase γ-Mo_2_N. Diffraction pattern of the Mo-(0.4N) coating shows three broad peaks, the maxima of which correspond to the planes (200), (111), and (220) of the γ-Mo_2_N phase. After decomposing a peak into its components, a broadened peak corresponding to α-Mo (110) is also identified.

An increase in the nitrogen flow rate in the reaction chamber from 0.4 sccm to 0.6 sccm led to an increase in the intensity of the reflections, corresponding to the γ-Mo_2_N phase. This proves the increase in the volume share of the γ-Mo_2_N phase in the coating. Additionally, the Mo- (0.6N) coating diffractogram shows a shift of the peaks, corresponding to planes (111), (200), and (220) of the γ-Mo_2_N phase, towards lower angular values. This is due to the occurrence of the compressive stresses in the coating. The presence of compressive stresses in metal nitride coatings produced in the PVD process is confirmed by the authors of [10,19,26,33,34]. The increase of stresses in the molybdenum nitride coatings because of increased nitrogen flow is confirmed by the authors of the work [11,17]. It was found that in the Mo-(0.6N) coating, the average crystallite size of the α-Mo phase was approximately two times larger than that of the γ-Mo_2_N phase (Table 2).

### 3.3. The Structure of the Coatings

**Figure 2:**
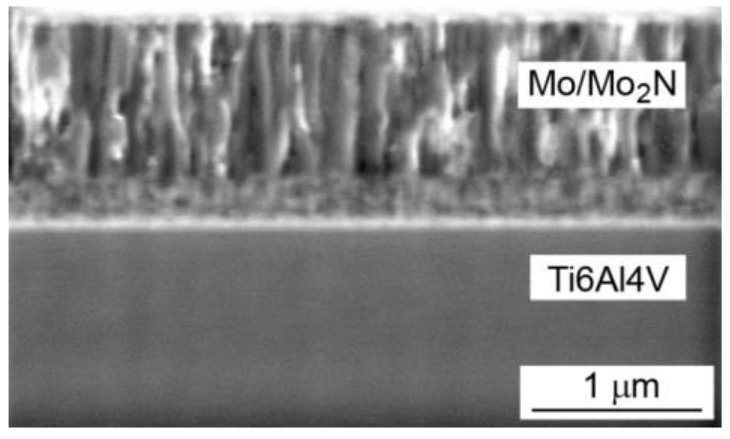
SEM image on the cross-section of the Mo-(0.4N) coating.

Microscopic examinations allowed to determine morphology of the coatings, phase composition and the size of the phase crystallites. Mo-Mo_2_N coatings had a columnar structure (Figure 2). The columns were perpendicular to the substrate. In the single-phase Mo and Mo-(0.2N) coatings, the diameter of the columnar crystals was in the range of 100 nm to 150 nm. With the higher proportions of nitrogen in the deposition process, the columns in the Mo-(0.4N) and Mo-(0.6N) two-phase coatings had a smaller diameter, in the range of 50 nm to 80 nm.

**Figure 3:**
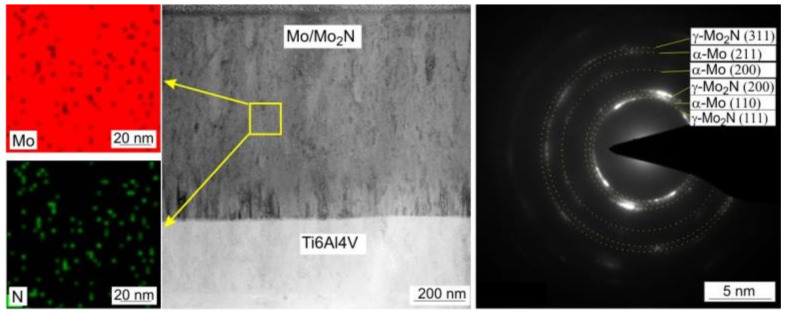
STEM image of Mo-N (0.6) coating in the center, chemical composition maps of N (green) and Mo (red) on the left, SAED diffraction on the right.

**Figure 4:**
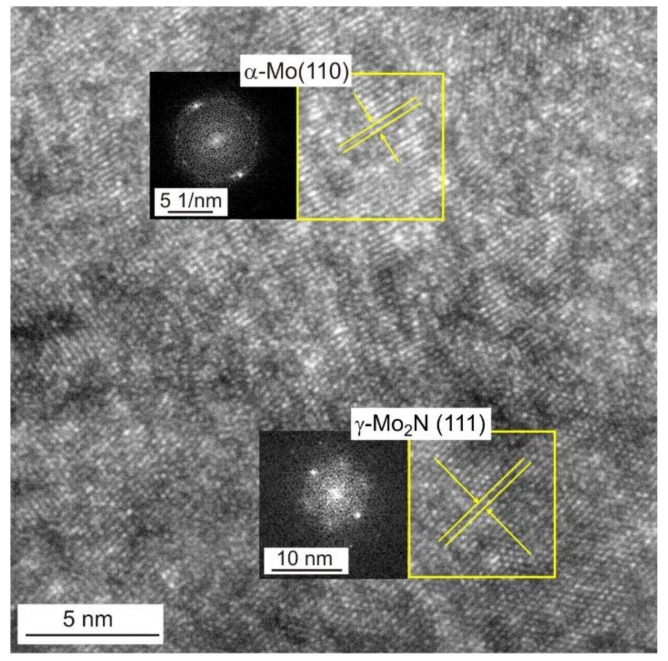
HRTEM image of Mo-N(0.6) coating with FFT transformations from areas marked by yellow frames. Visible crystallites of α-Mo (110) and γ-Mo_2_N (111).

Figure 3 shows STEM image of Mo-0.6N coating’s cross-section in the bright field with visible columnar crystals. EDS chemical composition maps confirmed the composite structure of the coating. Molybdenum nitride particles (visible in green, Figure 3) with sizes from 3 nm to 10 nm are evenly distributed in the molybdenum matrix, Figure 3. The SAED diffraction pattern reveals the preferred orientation of the molybdenum crystals (110). Additionally, the γ-Mo_2_N nitride grains show texture, they are oriented in the preferred direction (111) and (200) as evidenced by the partial rings in the electron diffraction pattern. In the Mo-(0.4N) coatings, crystallites of the α-Mo(110) and the γ-Mo_2_N (111) phases were identified, while in the Mo and Mo-(0.2N) coatings only the crystallites of the α-Mo (110) phase were present. A high-resolution transmission electron micrograph (HRTEM) and the corresponding FFT transformation images from the columnar crystal regions in the Mo-(0.6N) coating are shown in Figure 4. The FFT images were used to determine the interplanar distances in the marked crystallites. In the single crystallites, the planes (110) of the α-Mo matrix and (111) of the γ-Mo_2_N reinforcing phase were indexed.

### 3.4. Analysis of the Geometric Structure of the AFM Surface

**Figure 5:**
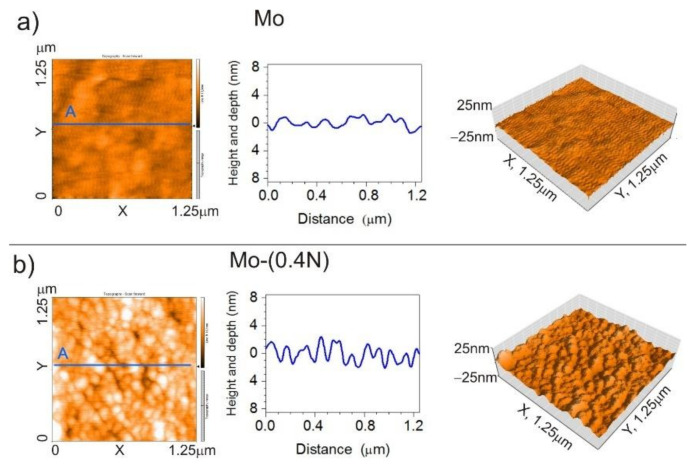
AFM images 2D, cross-section curve according to line A, images 3D of (**a**) Mo coating, (**b**) Mo-(0.4N) coating.

The geometrical structure of the produced coatings was examined using an atomic force microscope (AFM). During measurement, area of 1.25 μm **×** 1.25 μm was scanned (Figure 5). The Mo-Mo_2_N nanocomposite coatings had a homogeneous surface with low roughness Ra in the range of 1.1 nm to 1.8 nm. In the adopted range of nitrogen flow in the chamber (0–0.6) sccm, the trend of nitrogen influence on the roughness of the coating cannot be clearly established. The roughness parameters are presented in Table 3.

Geometrical structure of the surface determined by the AFM confirms columnar structure of the coating. Changes in topography correspond to the size of the columnar grains. Columnar grains visible on the surface with the growth direction perpendicular to the substrate have a diameter ranging from 100 nm to 200 nm in Mo (Figure 5a) and Mo-(0.2N) coatings. In the Mo-(0.4N) (Figure 5b) and Mo-(0.6N) coatings, with nitrogen concentrations of 12.1 at.% and 18.6 at.%, the diameter of the column grains was from 50 nm to 80 nm. It should be emphasized that the obtained coatings were homogeneous in terms of the diameter of the columns. Values obtained during the observation with the AFM microscope were consistent with the results obtained during the observation with the SEM microscope (Figure 2) and TEM (Figure 3). In the case of hard coatings, low surface roughness reduces the friction coefficient and the wear of the coating [35,36].

### 3.5. Analysis of Hardness, Young’s Modulus, and Adhesion to the Substrate

**Figure 6:**
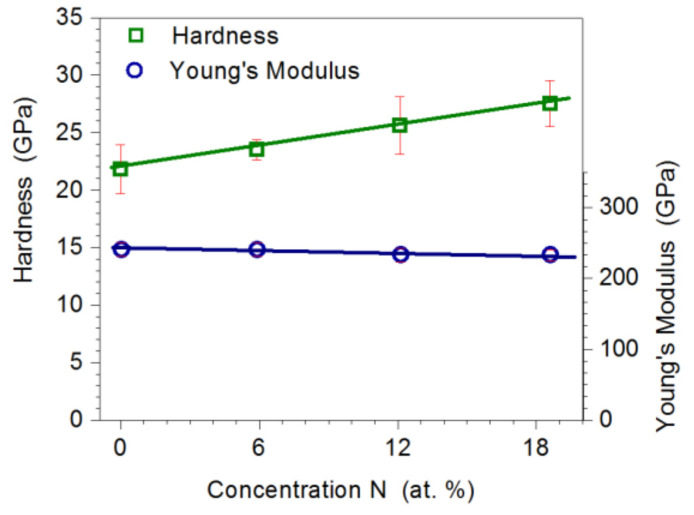
Influence of nitrogen concentration in the coating on the hardness and Young’s modulus of Mo-Mo_2_N coatings.

It was found that the increase in nitrogen concentration in the coating from 0 to 18.6 at.% causes an increase in its microhardness from 22.3 GPa to 27.5 GPa (Figure 6). The proportional increase in the hardness in the Mo-Mo_2_N coatings is caused by the increasing share of the hard Mo_2_N phase, with average crystallite sizes ranging from 7.1 nm to 10.4 nm and strengthening, due to the fragmentation of the matrix structure α-Mo. The increase in hardness is also due to the creation of favorable compressive stresses, which is indicated by the shift of the peaks on the XRD diffractograms.

Regardless of the nitrogen concentration in Mo-Mo_2_N coatings, Young’s modulus was in the range of 230 GPa to 240 GPa. Standard deviation in the measurements of hardness did not exceed 10% of the average value of the measurement and, in the measurements of Young’s modulus, 5% of the average value. It can be concluded that the coating growth process was stable and provided coatings with homogeneous mechanical properties.

According to [37,38], increase of the hardness of coatings containing molybdenum nitrides is caused by the formation of covalent bonds in the Mo-N phases and grain refinement. In turn, the authors of [39] explain the increase in the hardness of the coating by the formation of columnar crystals. According to Musil [40], there are three mechanisms responsible for the increase in hardness: plastic deformation caused by dislocation slip, microstructure of materials, and the forces of cohesion between atoms. Plastic deformation caused by dislocation slip dominates in materials with crystallite sizes d > 10 nm. On the other hand, the microstructure is dominant in materials with crystallites below d ≤ 10 nm. Hardness of γ Mo_2_N nitride coatings, reported in the literature, ranges from 18 GPa for coatings deposited by DC magnetron sputtering [41] to 38 GPa for coatings produced by means of cathodic arc evaporation [42]. Hardness of the produced coatings was within the range of the values reported in most of the literature references [10,13,23,24,41,42]. In some literature, for example [14], you can find hardness values that differ significantly from those obtained in this work. It should be noted, however, that those measurements were for much thicker coatings and were obtained with other indenters and at much higher loads.

**Figure 7:**
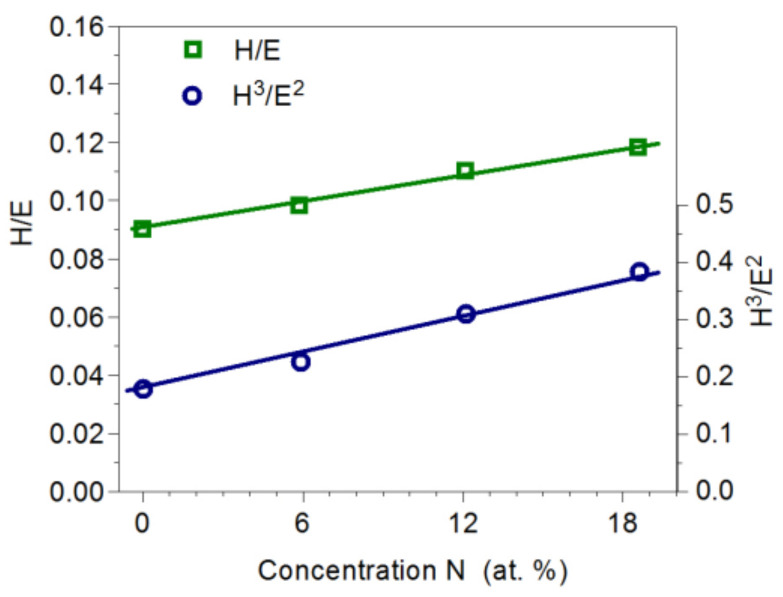
Influence of nitrogen concentration in Mo-Mo_2_N on the H/E and H^3^/E^2^ coefficients.

The H/E quotient carries information about resistance to damage during elastic deformations and the H^3^/E^2^ coefficient about resistance to plastic deformation. High values of the H/E and H^3^/E^2^ ratios ensure favorable tribological properties. Therefore, in the production of coatings, high hardness and low Young’s modulus should be sought. The H/E and H^3^/E^2^ ratios in the tested coatings were favorable. With an increase in nitrogen concentration in the coating from 0 to 18.6 at%, the H/E ratio changed from 0.090 to 0.118, while the H^3^/E^2^ ratio changed from 0.178 to 0.383 (Figure 7). Favorable values of the H/E and H^3^/E^2^ coefficients result from the relatively low Young’s modulus (230–240) GPa of the coating. Values of Young’s modulus for Mo_2_N nitride reported in the literature range from 380 GPa to 400 GPa [10,23].

**Figure 8:**
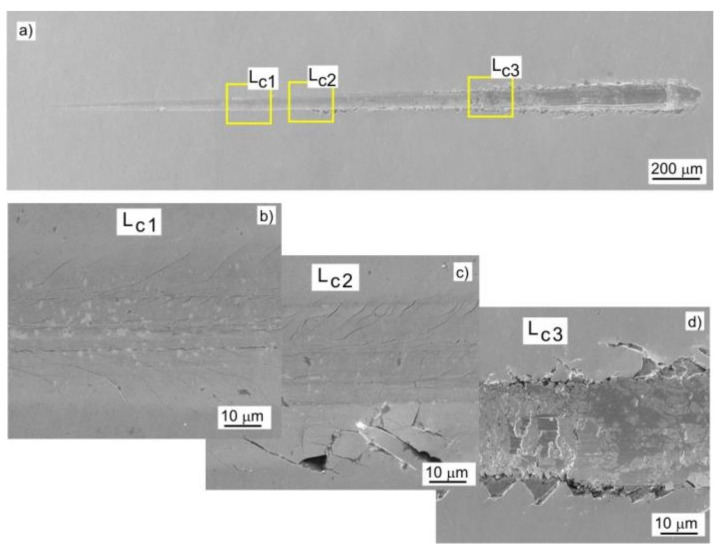
SEM image of a scratch on the Mo-(0.6N) coating: (**a**) view of the scratch, (**b**) cohesive longitudinal and angular cracks, (**c**) angular cracks and coating delamination at the bottom edge of the scratch, and (**d**) coating delamination along the entire width of the scratch.

**Figure 9:**
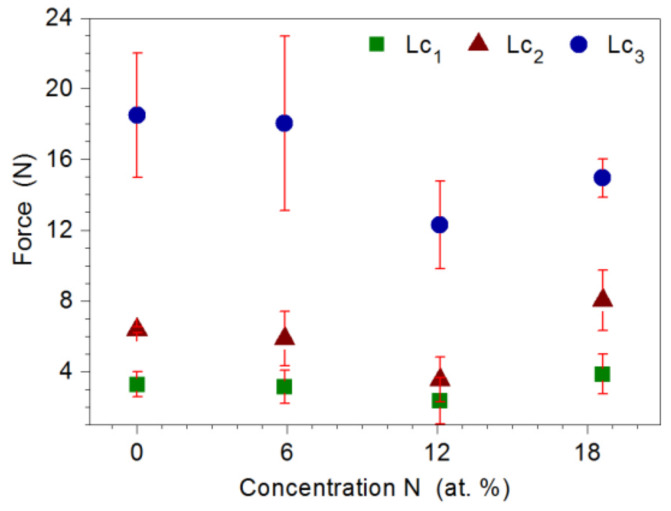
The values of the critical loads Lc_1_, Lc_2_, and Lc_3_, in relation to the nitrogen concentration in the Mo-Mo_2_N coating.

Adhesion of the coating to the substrate was determined by the scratch test method. Based on the analysis of changes in the value of friction force, acoustic emission signal and microscopic observations, the critical loads Lc_1_, Lc_2_, and Lc_3_ were determined, at which cohesive cracking, adhesive cracking, and coating delamination from the substrate occurred, respectively. Figure 8 shows a SEM image of the Mo-(0.6N) coating after the scratch test. Initially, cohesive angular and longitudinal cracks appeared in the coatings, which gradually turned into transverse cracks along the entire width of the scratch (Figure 8b). The first adhesive cracks, in the form of loosening and lifting of the coating from the substrate, were observed along the edge of the scratch (Figure 8c). In the last stage of coating destruction, the coating was delaminated from the substrate over the entire width of the scratch (Figure 8d). The highest loads of the Lc_1_ and Lc_2_ were recorded for the Mo-(0.6N) coating with the highest nitrogen content of 18.6 at.%. This proves high adhesion of the coating to the substrate. In coatings with nitrogen concentration of 12.1 at.%, the lowest values of all critical loads were recorded (Figure 9). Single-phase Mo, Mo-(0.2) coatings, are characterized by comparable adhesion. In the case of two-phase coatings Mo-(0.4N), Mo-(0.6), coating with the higher nitrogen content shows better adhesion. Mechanism of coating destruction during the scratch test for all Mo-Mo_2_N coatings was similar. 

### 3.6. Tribological Properties of Coatings

Tribological properties were determined for the Mo-Mo_2_N nanocomposite coatings and the Ti6Al4V substrate with a counter-sample—Al_2_O_3_ ball. In the area of the contact between the ball and the coating, stress σ_s_, determined according to Hertz’s formula [43], was 147 kPa for F = 1 N, 185 kPa for F = 2 N, and 251 kPa for F = 5 N. Due to insignificant differences in Young’s modulus of the tested coatings, the stress value (σ_s_) depended mainly on the load.

**Figure 10:**
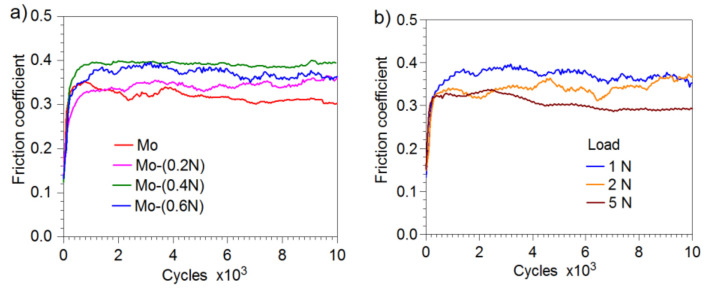
Friction coefficient: (**a**) Mo-Mo_2_N coatings produced at various nitrogen flow rates, load 1 N, (**b**) Mo- (0.6N) coatings at various loads.

After the first phase of the friction test, in which the ball performed 200–400 cycles (Figure 10), friction conditions stabilized. After this period, value of the friction coefficient was in the range from 0.3 to 0.4, depending on the load and the tested coating.

In the two-phase coatings Mo-(0.4N) and Mo-(0.6N), higher values of friction coefficient were observed than in the coatings with a single-phase structure Mo and Mo-(0.2N). The maximum and the average values of the friction coefficient for the various variants of the applied loads and coatings are given in Table 4. In Mo-Mo_2_N coatings, with increasing load, the average and maximum values of the friction coefficient slightly decrease. Reduction of the friction coefficient, with increasing load in the friction node, is confirmed in the works [44,45,46].

Mo-Mo_2_N nanocomposite coatings had a lower friction coefficient, compared to titanium nitrides [24,34,42], niobium nitrides [47,48,49], chromium nitrides [34,44,50], and zirconium nitrides [51,52]. Mo-Mo_2_N coatings produced by magnetron sputtering also had a lower friction coefficient, by about 0.1-0.2, compared to Mo-N coatings produced by the IBAD ion-beam-assisted deposition technology [15], as well as Mo-N coatings deposited under glow discharge conditions [14].

The maximum value of the friction coefficient determined during the tribological testing of the Ti6Al4V substrate was 0.6 and 0.5 with a load of 1 N and 5 N, respectively.

Tribological wear of the coating was characterized by the coefficient k, determined according to the relationship: k = V/Fs, where: k—wear factor, V—volume of material removed [m^3^], F—load in the friction node [N], s—friction path [m].

**Figure 11:**
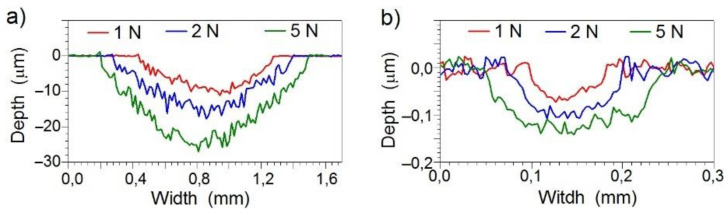
Profiles made along a line perpendicular to the friction trace: (**a**) for the Ti6Al4V substrate, (**b**) for the Mo-(0.6N) coating.

To calculate wear factor for a coating, measurements of the friction trace profile were performed along the line perpendicular to the direction of friction. Sample profiles for the Ti6Al4V substrate and Mo-(0.6 N) coating are shown in Figure 11. The substrate was characterized by a low wear resistance. The depth of the friction trace varied from 9 µm (F = 1 N) to 25 µm (F = 5 N). The depth of the friction trace in the tested Mo-Mo_2_N coatings ranged from 0.07 µm (F = 1 N) to 0.14 µm (F = 5 N) and was 20–30 times smaller than in the base material.

**Figure 12:**
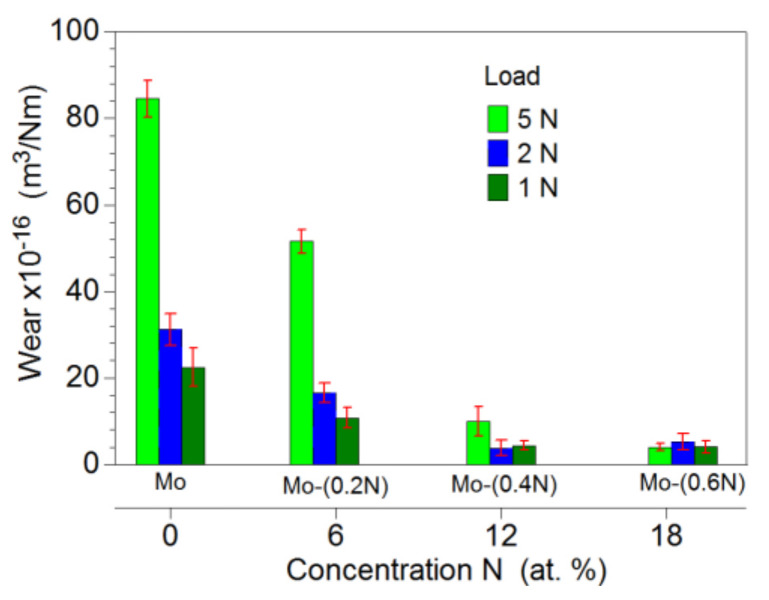
Coefficient of tribological wear of Mo-Mo_2_N coatings.

The Mo-Mo_2_N nanocomposite coatings under the evaluation were characterized by a low wear coefficient. With the increase in nitrogen concentration in the nanocomposite coating, there was a clear improvement in its resistance to tribological wear (Figure 12). The wear coefficient of the two-phase Mo-(0.4N) and Mo-(0.6N) coatings for the 5 N load was, respectively, 10 × 10^−16^ m^3^/N∙m and 4.0 × 10^−16^ m^3^/N∙m. For the single-phase Mo and Mo-(0.2N) coatings, the wear coefficient was 84.5 × 10^−16^ m^3^/N∙m and 51.6 × 10^−16^ m^3^/N∙m, respectively. The wear factor determined for the Ti6Al4V substrate was three orders of magnitude higher, in relation to the Mo-(0.6) coating. In the case of the Mo-(0.6) coating, the load force of the friction junction does not affect the wear factor.

**Figure 13:**
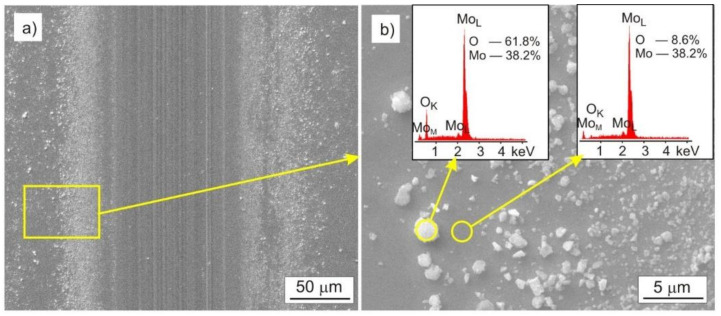
SEM image of the Mo coating after the tribological test, performed with the load F = 1 N: (**a**) trace of friction, (**b**) molybdenum oxides displaced beyond the trace of friction.

In the tested Mo-Mo_2_N coatings, two main wear mechanisms can be distinguished, one as a result of abrasive wear and another as a result of oxidation. In the case of the Mo coating, the abrasive wear mechanism dominated. The effects of this type of wear are numerous scratches parallel to the direction of ball travel. The tribochemical oxidation of Mo also took place in the friction zone, as a result of which, molybdenum oxides were formed on the surface. Molybdenum oxides were being moved beyond the friction area. Figure 13a shows oxides with a diameter ranging from 0.2 µm to 2 µm, located mainly on the left and right side of the friction trace. Molybdenum has a rich chemistry because it has oxidation states ranging from −II to + VI and coordination numbers from 0 to 8 [53]. Changes in the oxidation level and coordination number of the element determine the reactions taking place on the surface of metallic molybdenum.

The type and proportion of surface molybdenum oxides depends on the oxidation conditions, such as the type of medium (air, water vapor), as well as temperature and pressure. The main passivation oxide in the air environment is MoO_2_ [54]. Under the conditions of the tribological test, oxides are inevitably formed on the molybdenum surface, and moving the Al_2_O_3_ ball across the coating reveals the unoxidized molybdenum surface and contributes to the intensification of the oxidation process. The EDS analysis performed in the area of the oxide shows the presence of oxides chemically close to MoO_2_ oxide, Figure 13b.

**Figure 14:**
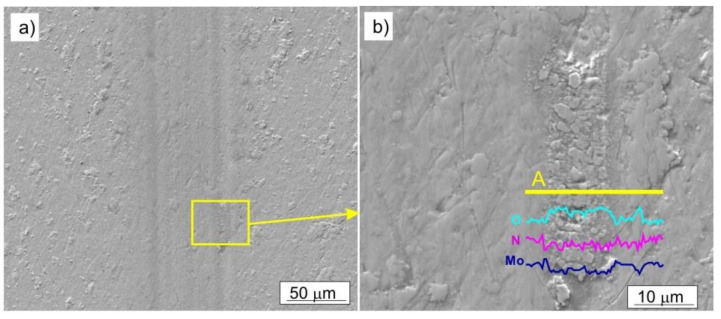
SEM image of the Mo-(0.4N) coating after the tribological test, performed with the load F = 2 N: (**a**) trace of friction, (**b**) molybdenum oxides smeared in the friction area, change of concentration of O, N, Mo along the line A.

In the two-phase Mo-Mo_2_N coatings, an abrasive wear and oxidation processes took place; additionally, there was a material transfer between the sample and the counter-sample. No sharp-edged scratches were observed on the surface of the coatings. The resulting molybdenum oxides were smeared in the area of friction. (Figure 14). An increase in oxygen concentration, in the area of friction trace, is confirmed by the EDS analysis, performed along a line perpendicular to the direction of ball movement, Figure 14b. Due to the presence of the α-Mo phase in Mo-Mo_2_N nanocomposite coatings, one should expect oxides, which form as a result of oxidation of this phase. Nevertheless, the oxidization of molybdenum nitrides is also possible.

Frictional oxidation of hard coatings consisting of nitrides of transitional metals containing molybdenum were reported by Yang et al. [8], Xu et al. [29], Polcar et al. [55], and Woydt et al. [56]. According to Solak and others [57], the process of oxidation of Mo-N coatings begins at the temperature of 350–400°C and proceeds, according to the following reactions: Mo_2_N + 2O_2_ → 2MoO_2_ + 1/2N_2_; MoO_2_ + 1/2O_2_ → MoO_3_. The value of the temperature in the friction zone can be estimated from the equation [4]: ΔT = 0.25 μFv/[(k_1_ + k_2_) a], where μ is the friction coefficient, F is the normal load, v is the sliding speed, k_1_ and k_2_ are the thermal conductivities of the coating and the Al_2_O_3_ ball, and a is the contact radius of the real contact area (a = (F/πH)^0.5^, where H—hardness of the coating). Assuming the averaged coefficient k_1_ = 130 W/m·K, k_2_ = 35 W/m·K of the Mo-Mo_2_N coating, and the maximum load on the friction node, the estimated temperature in the friction junction is ΔT = 75 °C. Because ΔT < 350 °C, the formation of molybdenum oxide at a lower temperature is more likely a result of the reaction [57]: 2Mo_2_N + 12H_2_O → 4MoO_3_ + 2NH_3_ + 9H_2_.

Molybdenum oxides present in the friction area effectively reduce the friction coefficient.

**Figure 15:**
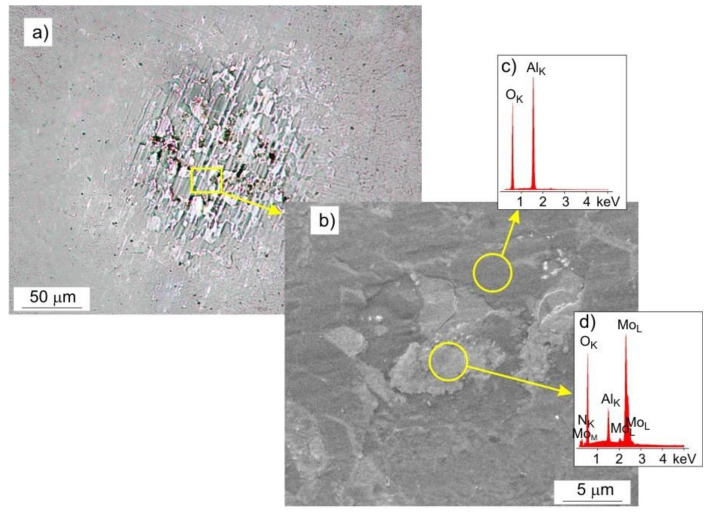
Surface of the Al_2_O_3_ ball after the friction test with the Mo-(0.4N) coating under a load of 1 N, (**a**) LM image, (**b**) SEM image, (**c**,**d**) EDS spectrograms.

Chemical composition analysis (EDS) showed that, during the tribological test, some of the coating material was transferred to the surface of the Al_2_O_3_ ball, Figure 15. In the areas of contact between the ball and the coating, the presence of smeared oxides, arranged in the direction of travel, was found (Figure 15a), while outside of the contact area, there were mainly spheroidal oxides. Based on the EDS analysis, it was determined that they were mainly molybdenum oxides, Figure 15c,d. No scratches were observed on the surface of the ball in the place of friction, Figure 15b.

During the examination of the Ti6Al4V substrate, oxidation of the alloy and material transfer to the ball were observed. In the first stage of the test, the oxide layer was removed from the surface of the alloy. A similar wear mechanism of a titanium alloy was observed by Tang et al. [14]. In the triboxidation process, hard TiO_x_ oxides were formed and ball roughness increased, which led to a much greater wear of the alloy, compared to the samples with Mo-Mo_2_N protective coatings.

## 4. Conclusions

A homogeneous, nanocomposite coating, consisting of the α-Mo matrix and γ-Mo_2_N reinforcing phases, was developed and produced in the magnetron sputtering process. The coating has a columnar structure, consisting of the α-Mo phase crystallites with the (110) orientation and γ-Mo_2_N with the (111) and (200) orientations. The mean size of the α-Mo phase crystallites was 29.4 nm and of the γ-Mo_2_N phase was 10.4 nm.

The influence of the nitrogen flow rate in the magnetron sputtering process on its content in the coating and on the formation of the γ-Mo_2_N phase was determined. The γ-Mo_2_N phase was formed at a nitrogen flow rate ranging from 0.4 sccm to 0.6 sccm.

Increasing the nitrogen flow rate increases the nitrogen concentration in the coating and leads up to the refinement of the columnar crystals.

The two-phase α-Mo + γ-Mo_2_N coating, containing 18.6 at.% nitrogen, produced at a nitrogen flow rate of 0.6 sccm, has the most favorable tribological properties. The coating has a hardness of 27.5 GPa and a low Young’s modulus of 223 GPa, ensuring the ratios of H/E = 0.12 and H^3^/E^2^ = 0.38. It is characterized by a low friction coefficient μ = 0.31 and coefficient of wear k = 4.0 × 10^−16^ m^3^/N∙m, has a low surface roughness below 1.7 nm, and the best adhesion to the substrate.

The dominant wear mechanism of the Mo-Mo_2_N coatings was abrasive wear and oxidation wear. In conclusion, it should be stated that the nanocomposite Mo-Mo_2_N coating is a prospective material for machine and device elements made of Ti6Al4V alloy operating in dry friction conditions.

## Figures and Tables

**Table 1:** Deposition parameters of Mo-Mo_2_N coatings.

Coating Designation	Type of Coating	Vacuum	Flow Rate Ar	Flow Eate N_2_	Duration
		[Pa]	[Sccm]	[Sccm]	[s]
Mo	Single-phase	0.53	2.5	-	1440
Mo-(0.2N)	Single-phase	0.53	2.5	0.2	1080
Mo-(0.4N)	Nanocomposite	0.53	2.5	0.4	1080
Mo-(0.6N)	Nanocomposite	0.56	2.5	0.6	1080

**Table 2:** Thickness, chemical composition, phase composition and crystallite size of coatings.

Coating Designation	Average Coating Thickness [μm]	Average Mo Concentration in the Coating [at.%]	Average N Concentration in the Coating [at.%]	Phase Composition	Average Crystallite Size	
					α-Mo [nm]	γ-Mo_2_N [nm]
Mo	1.33	100	0	α-Mo	18.5	-
Mo-(0.2N)	1.22	94.1 ± 1.6	5.9 ± 0.5	α-Mo	2.4	-
Mo-(0.4N)	1.13	87.9 ± 1.8	12.1 ± 0.8	α-Mo,	28.1	7.1
				γ-Mo_2_N		
Mo-(0.6N)	0.98	81.4 ± 2.9	18.6 ± 1.3	α-Mo,	29.4	10.4
				γ-Mo_2_N		

**Table 3:** Roughness parameters of Mo-Mo_2_N coatings.

Sample	Average Values of Roughness Parameters	
	Ra, nm	RMS, nm
Mo	1.4 ± 0.3	1.7 ± 0.3
Mo-(0.2N)	1.1 ± 0.3	1.5 ± 0.4
Mo-(0.4N)	1.8 ± 0.4	2.4 ± 0.4
Mo-(0.6N)	1.3 ± 0.2	1.7 ± 0.3

**Table 4:** Average and maximum value of the friction coefficient of the system: Mo-Mo_2_N coating—Al_2_O_3_ ball.

Coating	Friction Coefficient	Load_,_ F		
		1 N	2 N	5 N
		Friction Coefficient, μ		
Mo	average	0.31 ± 0.03	0.34 ± 0.03	0.31 ± 0.04
	max.	0.40	0.52	0.38
Mo-(0.2N)	average	0.34 ± 0.03	0.28 ± 0.05	0.27 ± 0.05
	max.	0.39	0.31	0.32
Mo-(0.4N)	average	0.39 ± 0.03	0.35 ± 0.03	0.29 ± 0.02
	max.	0.43	0.39	0.33
Mo-(0.6N)	average	0.38 ± 0.02	0.34 ± 0.03	0.31 ± 0.02
	max.	0.40	0.38	0.36

## Data Availability

Data sharing not applicable.

## References

[1] Bylica A., Sieniawski J. (1985). Titanium and Its Alloys.

[2] Philip J.T., Mathew J., Kuriachen B. (2019). Tribology of Ti6Al4V: A review. Friction.

[3] Budzynski P., Youssef A.A., Sielanko J. (2006). Surface modification of Ti–6Al–4V alloy by nitrogen ion implantation. Wear.

[4] Chen W., Hu T., Hong Y., Zhang D., Meng X. (2020). Comparison of microstructures, mechanical and tribological properties of arc-deposited AlCrN, AlCrBN and CrBN coatings on Ti-6Al-4V alloy. Surf. Coat. Technol..

[5] Gao Z., Zhang Z., Zhang X., Kulczyk-Malecka J., Liu H., Kelly P., Withers P.J., Xiao P. (2020). A conformable high temperature nitride coating for Ti alloys. Acta Mater..

[6] Yang K., Xian G., Zhao H., Fan H., Wang J., Wang H., Du H. (2015). Effect of Mo content on the structure and mechanical properties of TiAlMoN films deposited on WC-Co cemented carbide substrate by magnetron sputtering. Int. J. Refract. Hard Met..

[7] Xu J., Ju H., Yu L. (2012). Effects of Mo content on the microstructure and friction and wear properties of TiMoN films. Acta Metall. Sin..

[8] Yang Q., Zhao L.R., Patnaik P.C., Zeng X.T. (2006). Wear resistant TiMoN coatings deposited by magnetron sputtering. Wear.

[9] Hahn R., Koutná N., Wójcik T., Davydok A., Kolozsvári S., Krywka C., Holec D., Bartosik M., Mayrhofer P.H. (2020). Mechanistic study of superlattice-enabled high toughness and hardness in MoN/TaN coatings. Commun. Mater.

[10] Xu J., Ju H., Yu L. (2014). Microstructure, oxidation resistance, mechanical and tribological properties of Mo-Al-N films by reactive magnetron sputtering. Vacuum.

[11] Stöber L., Konrath J.P., Haberl V., Patocka F., Schneider M., Schmid U. (2016). Nitrogen incorporation in sputter deposited molybdenum nitride thin films. J. Vac. Sci. Technol. A.

[12] Sergevnin V.S., Blinkov I.V., Volkhonskii A.O., Belov D.S., Kuznetsov D.V., Gorshenkov M.V., Skryleva E.A. (2016). Wear behaviour of wear-resistant adaptive nano-multilayered Ti-Al-Mo-N coatings. Appl. Surf. Sci..

[13] Suszko T., Gulbiński W., Jagielski J. (2005). The role of surface oxidation in friction processes on molybdenum nitride thin films. Surf. Coat. Technol..

[14] Tang B., Wu P.Q., Li X.Y., Fan A.L., Xu Z., Celis J.P. (2004). Tribological behavior of plasma Mo-N surface modified Ti-6Al-4V alloy. Surf. Coat. Technol..

[15] Zhu X., Yue D., Shang C., Fan M., Hou B. (2013). Phase composition and tribological performance of molybdenum nitride coatings synthesized by IBAD. Surf. Coat. Technol..

[16] Gilewicz A., Warcholinski B., Murzynski D. (2013). The properties of molybdenum nitride coatings obtained by cathodic arc evaporation. Surf. Coat. Technol..

[17] Stöber L., Konrath J.P., Krivec S., Patocka F., Schwarz S., Bittner A., Schneider M., Schmid U. (2015). Impact of sputter deposition parameters on molybdenum nitride thin film properties. J. Micromech. Microeng..

[18] Jauberteau I., Bessaudou A., Mayet R., Cornette J., Jauberteau J.L., Carles P., Merle-Méjean T. (2015). Molybdenum nitride films: Crystal structures, synthesis, mechanical, electrical and some other properties. Coatings.

[19] Kazmanli M.K., Ürgen M., Cakir A.F. (2003). Effect of nitrogen pressure, bias voltage and substrate temperature on the phase structure of Mo-N coatings produced by cathodic arc PVD. Surf. Coat. Technol..

[20] Klimashin F.F., Koutná N., Euchner H., Holec D., Mayrhofer P.H. (2016). The impact of nitrogen content and vacancies on structure and mechanical properties of Mo-N thin films. J. Appl. Phys..

[21] Bouaouina B., Besnard A., Abaidia S.E., Airoudj A., Bensouici F. (2018). Correlation between mechanical and microstructural properties of molybdenum nitride thin films deposited on silicon by reactive R.F. magnetron discharge. Surf. Coat. Technol..

[22] Pappacena K.E., Singh D., Ajayi O.O., Routbort J.L., Erilymaz O.L., Demas N.G., Chen G. (2012). Residual stresses, interfacial adhesion and tribological properties of MoN/Cu composite coatings. Wear.

[23] Zhang G., Fan T., Wang T., Chen H. (2013). Microstructure, mechanical and tribological behavior of MoN_x_/SiN_x_ multilayer coatings prepared by magnetron sputtering. Appl. Surf. Sci..

[24] Zhang G., Wang T., Chen H. (2015). Microstructure, mechanical and tribological properties of TiN/Mo_2_N nanomultilayer films deposited by magnetron sputtering. Surf. Coat. Technol..

[25] Chen X., Du Y., Chung Y.W. (2019). Commentary on using H/E and H^3^/E^2^ as proxies for fracture toughness of hard coatings. Thin Solid Films.

[26] Gilewicz A., Warcholinski B. (2015). Deposition and characterisation of Mo_2_N/CrN multilayer coatings prepared by cathodic arc evaporation. Surf. Coat. Technol..

[27] Yang Q. (2017). Wear resistance and solid lubricity of molybdenum-containing nitride coatings deposited by cathodic arc evaporation. Surf. Coat. Technol..

[28] Gu B., Tu J.P., Zheng X.H., Yang Y.Z., Peng S.M. (2008). Comparison in mechanical and tribological properties of Cr-W-N and Cr-Mo-N multilayer films deposited by DC reactive magnetron sputtering. Surf. Coat. Technol..

[29] Xu J., Ju H., Yu L. (2014). Influence of silicon content on the microstructure, mechanical and tribological properties of magnetron sputtered Ti-Mo-Si-N films. Vacuum.

[30] Beresnev V.M., Bondar O.V., Postolnyi B.O., Lisovenko M.O., Abadias G., Chartier P., Kolesnikov D.A., Borisyuk V.N., Mukushev B.A., Zhollybekov B.R. (2014). Comparison of tribological characteristics of nanostructured TiN, MoN, and TiN/MoN arc-PVD coatings. J. Frict. Wear.

[31] International Organization for Standardization (2015). Metallic Materials — Instrumented Indentation Test for Hardness and Materials Parameters — Part 1: Test Method.

[32] Oliver W.C., Pharr G.M. (1992). An improved technique for determining hardness and elastic modulus using load and displacement sensing indentation experiments. J. Mater. Res..

[33] Postolnyi B.O., Beresnev V.M., Abadias G., Bondar O.V., Rebouta L., Araujo J.P., Pogrebnjak A.D. (2017). Multilayer design of CrN/MoN protective coatings for enhanced hardness and toughness. J. Alloys Compd..

[34] Öztürk A., Ezirmik K.V., Kazmanli K., Ürgen M., Eryilmaz O.L., Erdemir A. (2008). Comparative tribological behaviors of TiN, CrN and MoNCu nanocomposite coatings. Tribol. Int..

[35] Shaha K.P., Pei Y.T., Martinez-Martinez D., De Hosson J.T.M. (2010). Influence of hardness and roughness on the tribological performance of TiC/a-C nanocomposite coatings. Surf. Coat. Technol..

[36] Gheisari R., Polycarpou A.A. (2018). Three-body abrasive wear of hard coatings: Effects of hardness and roughness. Thin Solid Films.

[37] Wang T., Zhang G., Ren S., Jiang B. (2017). Effect of nitrogen flow rate on structure and properties of MoN_x_ coatings deposited by facing target sputtering. J. Alloys Compd..

[38] Greczynski G., Tengstrand J., Lu O., Petrov I., Greene J.E., Hultman L. (2016). Nitrogen-doped: Bcc-Cr films: Combining ceramic hardness with metallic toughness and conductivity. Scr. Mater..

[39] Toyoda T., Sutou Y., Komiyama S., Ando D., Koike J., Wang M. (2016). Hardness and wear properties of Ti-Mo-C-N film. Mater. Trans..

[40] Musil J. (2012). Hard nanocomposite coatings: Thermal stability, oxidation resistance and toughness. Surf. Coat. Technol..

[41] Rudnik P.J., Graham M.E., Sproul W.D. (1991). High rate reactive sputtering of MoN_x_ coatings. Surf. Coat. Technol..

[42] Ürgen M., Eryilmaz O.L., Çakir A.F., Kayali E.S., Nilüfer B., Işik Y. (1997). Characterization of molybdenum nitride coatings produced by arc-PVD technique. Surf. Coat. Technol..

[43] Kot M. (2012). Contact mechanics of coating-substrate systems: Monolayer and multilayer coatings. Arch. Civ. Mech. Eng..

[44] Zhou F., Chen K., Wang M., Xu X., Meng H., Yu M., Dai Z. (2008). Friction and wear properties of CrN coatings sliding against Si_3_N_4_ balls in water and air. Wear.

[45] Stoyanov P., Strauss H.W., Chromik R.R. (2012). Scaling effects between micro- and macro-tribology for a Ti–MoS_2_ coating. Wear.

[46] Hudec T., Mikula M., Satrapinskyy L., Roch T., Truchlý M., Švec P., Huminiuc T., Polcar T. (2019). Structure, mechanical and tribological properties of Mo-S-N solid lubricant coatings. Appl. Surf. Sci..

[47] Ezirmik K.V., Rouhi S. (2014). Influence of Cu additions on the mechanical and wear properties of NbN coatings. Surf. Coat. Technol..

[48] Ju H., Xu J. (2015). Microstructure and tribological properties of NbN-Ag composite films by reactive magnetron sputtering. Appl. Surf. Sci..

[49] Wang T., Jin Y., Bai L., Zhang G. (2017). Structure and properties of NbN/MoN nano-multilayer coatings deposited by magnetron sputtering. J. Alloys Compd..

[50] Xingrun R., Qinying Z., Zhu H., Wei S., Jiangao Y., Hao C. (2018). Microstructure and Tribological Properties of CrN Films Deposited by Direct Current Magnetron Sputtering. Rare Met. Mater. Eng..

[51] Singh A., Kumar N., Kuppusami P., Prasanthi T.N., Chandramohan P., Dash S., Srinivasan M.P., Mohandas E., Tyagi A.K. (2012). Tribological properties of sputter deposited ZrN coatings on titanium modified austenitic stainless steel. Wear.

[52] Valerini D., Signore M.A., Tapfer L., Piscopiello E., Galietti U., Rizzo A. (2013). Adhesion and wear of ZrN films sputtered on tungsten carbide substrates. Thin Solid Films.

[53] Bard A.J., Parsons R., Jordan J. (1985). Standard Potentials in Aqueous Solution.

[54] Saji V.S., Lee C.W. (2012). Molybdenum, Molybdenum Oxides, and their Electrochemistry. ChemSusChem.

[55] Polcar T., Parreira N.M.G., Cavaleiro A. (2007). Tribological characterization of tungsten nitride coatings deposited by reactive magnetron sputtering. Wear.

[56] Woydt M., Skopp A., Dörfel I., Witke K. (1999). Wear Engineering Oxides/Antiwear Oxides. Tribol. Trans..

[57] Solak N., Ustel F., Urgen M., Aydin S., Cakir A.F. (2003). Oxidation behavior of molybdenum nitride coatings. Surf. Coat. Technol..

